# Washout and Awakening Times after Inhaled Sedation of Critically Ill Patients: Desflurane Versus Isoflurane

**DOI:** 10.3390/jcm10040665

**Published:** 2021-02-09

**Authors:** Philipp Daume, Johannes Weis, Hagen Bomberg, Martin Bellgardt, Thomas Volk, Heinrich V. Groesdonk, Andreas Meiser

**Affiliations:** 1 Department of Anesthesiology, Intensive Care Medicine and Pain Medicine, Saarland University Hospital Medical Center, 66421 Homburg, Germany; js.weis@t-online.de (J.W.); hagenbomberg@web.de (H.B.); Thomas.Volk@uks.eu (T.V.); Andreas.Meiser@uks.eu (A.M.); 2 Department of Anesthesiology and Intensive Care Medicine, St. Josef Hospital, Katholisches Klinikum Bochum, University Hospital, Ruhr-University of Bochum, 44791 Bochum, Germany; martin.bellgardt@rub.de; 3 Outcomes Research, Cleveland Clinic, Cleveland, OH 44195, USA; 4 Department of Intensive Care Medicine, Helios Clinic Erfurt, 99089 Erfurt, Germany; Heinrich.Groesdonk@helios-gesundheit.de

**Keywords:** inhaled sedation, volatile anesthetics, isoflurane, desflurane, intensive care unit (ICU) sedation

## Abstract

In recent years, inhaled sedation has been increasingly used in the intensive care unit (ICU). The aim of this prospective, controlled trial was to compare washout and awakening times after long term sedation with desflurane and isoflurane both administered with the Mirus™ system (TIM GmbH, Koblenz, Germany). Twenty-one consecutive critically ill patients were alternately allocated to the two study groups, obtaining inhaled sedation with either desflurane or isoflurane. After 24 h study sedation, anesthetic washout curves were recorded, and a standardized wake-up test was performed. The primary outcome measure was the time required to decrease the endtidal concentration to 50% (T50%). Secondary outcome measures were T80% and awakening times (all extremities moved, RASS −2). Decrement times (min) (desflurane versus isoflurane, median (1st quartile—3rd quartile)) (T50%: 0.3 (0.3–0.4) vs. 1.3 (0.4–2.3), log-rank test P = 0.002; P80%: 2.5 (2–5.9) vs. 12.1 (5.1–20.2), P = 0.022) and awakening times (to RASS −2: 7.5 (5.5–8.8) vs. 41.0 (24.5–43.0), P = 0.007; all extremities moved: 5.0 (4.0–8.5) vs. 13.0 (8.0–41.25), P = 0.037) were significantly shorter after desflurane compared to isoflurane. The use of desflurane with the Mirus™ system significantly shortens the washout times and leads to faster awakening after sedation of critically ill patients.

## 1. Introduction

The principle of anesthetic reflection permits efficient use of volatile anesthetics with common intensive care unit (ICU) ventilators [1]. In recent years, volatile anesthetics have been increasingly used for inhaled sedation of invasively ventilated critically ill patients [2]. They are referred to in National guidelines as alternative drugs for sedation, especially when deep sedation and rapid awakening are required [3,4,5].

**Figure 1:**
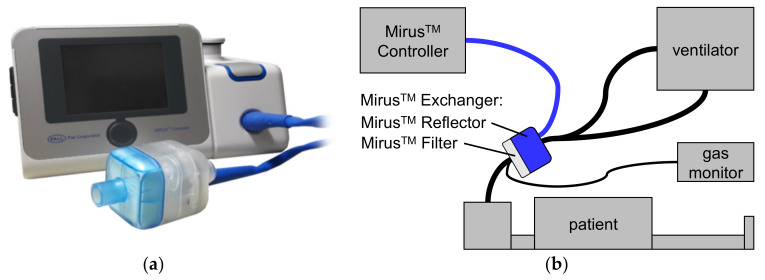
The MIRUS™ system (TIM GmbH, Koblenz, Germany). (**a**) control unit; (**b**) setup with a patient. The control unit is connected via a multi-lumen cable (blue line) with the Mirus™ Exchanger, interposed between ventilator hoses and the endotracheal tube of the patient. The Mirus™ Exchanger consists of two parts: The Mirus™ Filter represents a common heat moisture exchanger as well as a viral and bacterial filter. In the Mirus™ Reflector, volatile anesthetic is injected as saturated vapor and also reflected back to the patient; gas concentrations, airway pressure and flow are measured. In our study, an additional, external gas monitor was used.

The Mirus™ system (TIM GmbH, Koblenz, Germany) (Figure 1) was introduced in 2013 and comprises a gas and a ventilation monitor, along with an administration unit for isoflurane, sevoflurane, or desflurane [6]. The anesthetic is injected as saturated vapor into the breathing gas at the beginning of the inspiration. A control unit is connected via a multi-lumen cable with the Mirus™ Exchanger which comprises a total internal volume of 100 mL interposed between ventilator hoses and the endotracheal tube. The Mirus™ Exchanger includes a common heat moisture exchanger (HME) with viral and bacterial filter (Mirus™ Filter), which can be exchanged separately when spoiled or between patients. In the Mirus™ Reflector, the vaporized anesthetic is injected; gas is sampled for monitoring gas concentrations; airway pressure and flow are measured; and exhaled anesthetic is reflected back to the patient.

The blood/gas partition coefficient of desflurane is low, giving it better pharmacokinetic properties with less accumulation and faster wash-in and washout than isoflurane or sevoflurane. Many studies have shown faster awakening after desflurane anesthesia [7,8], especially in the obese [9], elderly [10,11] and after long lasting anesthesia [12]. It has been used for short term inhaled sedation of postoperative patients in the intensive care unit, allowing faster and more predictable awakening times compared to propofol [13].

We tested the hypothesis that volatile anesthetic washout is faster after 24 h of sedation with desflurane than after isoflurane, primarily assessed as the time required to reduce the end-tidal concentration to 50% (T50%). Secondarily, we hypothesized that times to 30%, 40%, 60%, 70%, and 80% reductions (T30%, T40%, T60%, T70%, and T80%) and awakening times (open eyes, squeeze hand, first extremity moved, all extremities moved, RASS -1, RASS -2) would also be shorter with desflurane. Additionally, we evaluated total anesthetic consumption of the Mirus™ system.

## 2. Experimental Section

We enrolled critically ill adults who were expected to require mechanical ventilation and sedation for at least 24 h. We excluded patients who were pregnant, started mechanical ventilation more than 48 h before the study, had tidal volumes less than 300 mL, had severe acute neurological illness or head injury, were deaf, were unable to follow simple commands, and were unable to communicate in German or English. We also excluded patients who had an expected survival time less than 24 h, who did not have an authorized legal representative, and those that had contraindications to volatile anesthetics such as personal or family history of malignant hyperthermia or halothane hepatitis.

Patients were included consecutively and were alternately allocated to desflurane (Suprane, Baxter Deutschland GmbH, Unterschleißheim, Deutschland) or isoflurane (Forene, Abbvie Deutschland GmbH und Co KG, Ludwigshafen, Deutschland), both applied with the Mirus™.

All patients were ventilated via endotracheal tube with an Evita 4 ventilator (Drägerwerk AG & Co. KGaA, Lübeck, Germany) in pressure-controlled mode or assisted with pressure support. Continuous propofol and remifentanil infusions provided sedation and analgesia before the sedation trial began.

The Mirus™ control unit was connected to the Mirus™ Exchanger (Figure 1). Anesthetic gas scavenging (Mirus™ ORS-Clean-Air, TIM GmbH, Koblenz, Germany) was connected to the expiratory port of the ventilator. The endotracheal tube was clamped and the standard HME (Humid-Vent Filter Compact S, Teleflex Medical GmbH, Kernen, Germany) was replaced by the Mirus™ Exchanger. A target concentration was initially set to 0.3 age-adjusted minimum alveolar anesthetic concentration (MAC) [14,15]. The propofol infusion was stopped, and the remifentanil infusion rate was halved. Minute ventilation was gradually reduced to let PaCO_2_ increase up to 60 mmHg in an effort to encourage assisted spontaneous breathing. If necessary, the remifentanil infusion was further decreased. Every two hours, the volatile anesthetic administration was adjusted to target Richmond Agitation and Sedation Scale [16] (RASS) Scores between −3 and −4.

After a planned sedation time of 24 ± 6 h, the endotracheal tube was clamped, and the Mirus™ Exchanger replaced by an HME. Gas monitoring continued using an external gas monitor (Vamos, Drägerwerk AG & Co. KGaA, Lübeck, Germany). Endtidal desflurane and isoflurane concentrations during washout were extracted from online breath by breath recordings. Primary outcome was the time required to decrease the endtidal concentration to 50% of the value when sedation was stopped (T50%).Other decrement times (T30%, T40%, T60%, T70%, and T80%) were also evaluated.

In parallel, a standardized awakening test was performed. Every minute, the patients were addressed with their names, asked to open their eyes, to squeeze their hand, and to move their right or left foot or hand. The sedation window ended once the patients had moved all extremities on command or after 60 min. The times to reach RASS-Scores of −2, −1 and 0 were also documented. Additionally, consumption of volatile anesthetics as registered by the Mirus™ system was recorded.

The collected data were processed with Excel (Microsoft Corporation, Redmond, WA, USA) and the statistical analysis was performed with SPSS Statistics (International Business Machines Corporation, Armonk, NY, USA). Continuous variables are expressed as means ± standard deviations or median [1st–3rd quartile] when data were not normally distributed. Testing for normal distribution was performed using Shapiro-Wilk test. Study groups were compared using two-sided unpaired t-tests for independent samples or Mann–Whitney’s U tests. Categorical variables are presented as numbers of patients and compared between groups using chi-square tests. Washout and awakening times were compared using log-rank tests. Statistical significance was accepted at two-sided significance level of 0.05.

An a priori power analysis was not possible because the statistical distribution of the primary outcome measure (T50%) was not known. During an interim analysis of 6 patients per group, the primary outcome measure showed a significant difference between groups (log-rank test: P = 0.036). Because of a very skewed distribution of data, a power analysis based on parametrical tests was not appropriate. With regard to the secondary outcome measures and to comply with other studies [17,18], the total number of patients was fixed at 10 patients in each group.

## 3. Results

**Figure 2:**
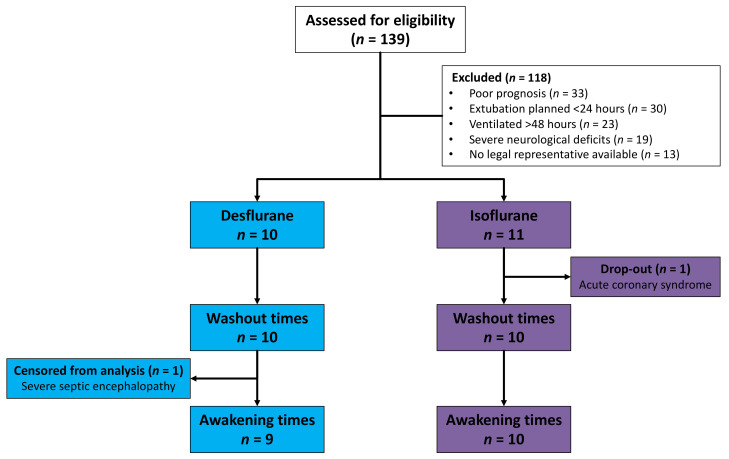
Flowchart of patients assessed for eligibility, allocated, and included in analysis. One patient did not show any signs of awakening during 60 min because of severe septic encephalopathy and was censored from the analysis of awakening times.

Between October 2016 and May 2017, 139 ICU patients in a German University Hospital were assessed for eligibility. A number of patients were excluded because of poor prognosis, because extubation was planned within 24 h, because they had been invasively ventilated for more than 48 h before possible inclusion, because of severe neurological deficits or because no legal representative was available (Figure 2). One patient allocated to the isoflurane group dropped out after developing acute coronary syndrome and being taken for coronary catheterization. Drug elimination was evaluated in ten patients in each group. One desflurane patient with severe sepsis and septic encephalopathy did not show any signs of awakening during 60 min. This patient was censored from the Kaplan–Meier analysis of awakening times.

Patients’ characteristics were similar in each anesthetic group (Table 1). Patients were severely ill as evidenced by high Simplified Acute Physiology Scores II (SAPS II) [19] and high Sepsis-related Organ Failure Assessment Scores (SOFA) [20] and poor oxygenation indices. Admission diagnoses, duration of application of the anesthetics, MAC fraction, RASS Scores and remifentanil dose did not differ significantly between the two groups. Six patients given desflurane and four given isoflurane were breathing spontaneously when sedation with volatile anesthetic began. The remaining patients soon started breathing spontaneously.

**Figure 3:**
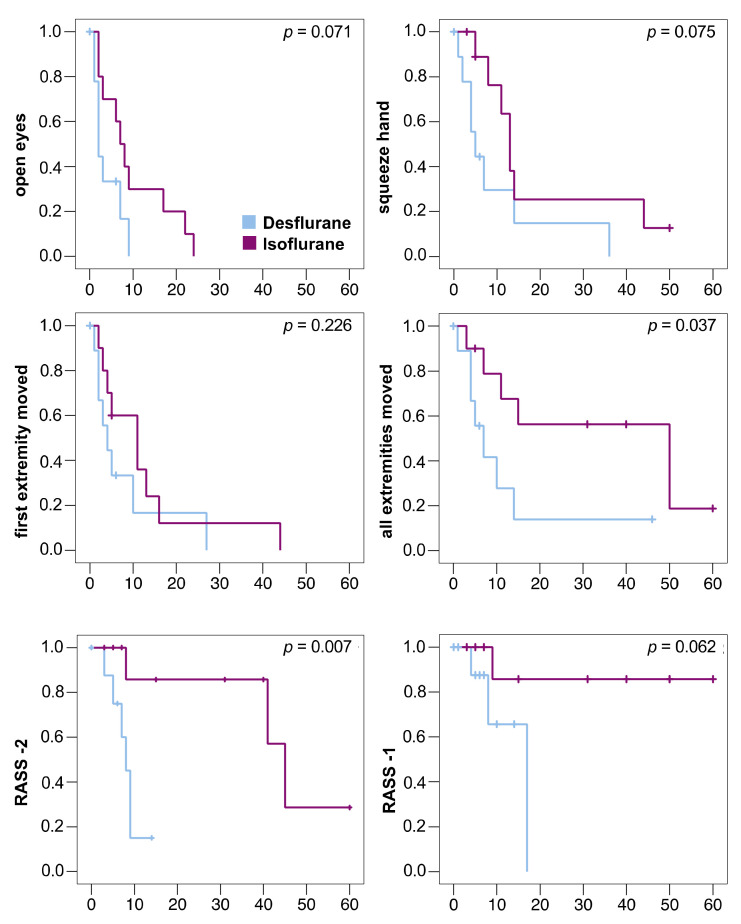
Kaplan–Meier diagrams of awakening times of 10 patients in each group. Patients were censored in case the sedation window had to be interrupted (vertical dashes). One desflurane patient with severe septic encephalopathy was censored at time point 0. All times in minutes, statistical comparison using log-rank test. RASS -2: Time to reach a Richmond Agitation Sedation Scale (RASS) score of −2; RASS -1: Time to reach a RASS score of −1. Only 4 patients reached RASS -1 (desflurane: 3 patients, isoflurane: 1 patient).

The primary outcome measure was reached in all patients. The sedation window had to be interrupted in 6 patients (desflurane/isoflurane: 2/4) before they moved all extremities because of bucking against the ventilator (0/1), high blood pressure (1/0), transport to diagnostic (0/1) or surgical procedures (1/0), or the end of 60 min observation time (0/2). These patients were censored at the respective time point in the Kaplan–Meier analysis (Figure 3). No patient died during the study.

Anesthetic washout was faster after desflurane compared to isoflurane (Table 2). All decrement times (T30%, T40%, T50%, T60%, T70%, and T80%) were significantly shorter after desflurane (Table 2). The times to reach RASS score −2 and until the patients were able to move all extremities on command were significantly shorter after desflurane compared to isoflurane. (Figure 3)

Consumption of desflurane per hour study sedation was 6.6-fold greater than that of isoflurane (29 ± 12 mL vs. 4 ± 3 mL). When related to MAC hours, consumption was 5.6 times that of isoflurane (61 ± 18 vs. 11 ± 3 mL).

## 4. Discussion

To our knowledge this is the first study to compare washout and awakening times after inhaled sedation of severely ill patients with desflurane and isoflurane. All measured decrement times and the times to move all extremities and to reach a RASS score of −2 were significantly shorter after desflurane compared to isoflurane. Besides significantly shorter times, it is of note that the interquartile ranges of the decrement times after desflurane were much smaller than after isoflurane (Table 2). In the ICU, a rapid and reliably predictable awakening is an advantage, as it shortens the time during which the patient needs close attention by staff in a sedation window.

Romagnoli evaluated the feasibility and safety of the Mirus™ system for sedation with sevoflurane in 62 postoperative patients for a median time of 3.3 h, and concluded that the Mirus™ was a promising and safe alternative for short term sedation with sevoflurane of ICU patients [21]. In contrast our patient group was more severely ill as evidenced by high SAPS II and SOFA Scores, study sedation was much longer, and we report the use of isoflurane, but also the use of desflurane with a reflection system in the ICU.

In a randomized controlled trial, Bellgardt et al. compared anesthetic washout, awakening times and therapy costs using 0.5 MAC desflurane, sevoflurane and isoflurane with the Mirus™ system in 30 postoperative patients. In accordance with our results, the study showed favorable kinetics for desflurane but also high desflurane consumption leading to high therapy costs [22]. While sedation time was comparable, the study population of patients after scheduled major surgery was not as severely ill as our patients and all could be extubated after stop of study sedation. To mention, for washout measurements the reflector was not removed from the breathing circuit.

In a case report, washout and awakening of a single patient were described in a sedation window after 24 h sedation with both drugs consecutively with very similar results [23].

In a study evaluating short term postoperative sedation, desflurane showed shorter and more predictable extubation times, as well as a quicker mental recovery compared to propofol. In a five-word memory test, patients after desflurane recalled significantly more words than patients after propofol [13]. Unfortunately, this could not be tested in our study, as our patients were too severely ill, could not be extubated, and needed continued sedation after the wake-up test.

When inhaled sedation is performed at a concentration just above MAC-awake [24], awakening and extubation (if possible) will be quick whatever volatile anesthetic is used. In our study, 50% decrement times are short after both anesthetics and differ little—only by about one minute. This small difference of T50% is statistically significant but unimportant for clinical practice. However, median 80% decrement times are more divergent (2.5 vs. 12.1 min). We consider it as an advantage, if the patients become fully conscious in a reasonably short time span, so that they can communicate and memorize information given. Then, the patients may be explained their situation, realize the circumstances, and stay calm. For this purpose, 80%, not 50%, decrement times are relevant.

It is a limitation of our study that the study team was not blinded during data collection. However, gas concentrations may be considered objective measurements and awakening was assessed using standardized questions. As this was a non-interventional study, patients were not randomized but were allocated alternately to the two study arms. This was an investigator-initiated trial with limited resources, and it was not our aim to perform a pharmaceutical study. The alternate treatment allocation allowed us to include two patients simultaneously, although we only had one device for desflurane and one for isoflurane. We minimized selection bias by including patients consecutively. Similar to other pharmacokinetic studies [17,18], we only included a small number of patients, which was enough to describe significant differences between the two anesthetics in this group of critically ill patients. As we included only very severely ill patients, most with sepsis and some with encephalopathy, not all patients did fully awake and none was extubated in the sedation window following the study sedation. Therefore, not all awakening times and no extubation times can be reported. On the other hand, it has been shown that inhaled sedation may be beneficial in patients with acute respiratory distress syndrome (ARDS) [25,26,27] and may be associated with a lower mortality [28] compared to intravenous sedation in severely ill patients. Therefore, our focus was on evaluating this new sedation method in those most severely ill patients that also may profit most.

Consumption of a volatile anesthetic during inhaled sedation is primarily determined by anesthetic losses through the reflector [29]. In a bench study, desflurane consumption with the Mirus™ was determined as 14.7 mL·h^−1^ when using conditions like in the present study (3.0 Vol% desflurane, 500 mL tidal volume), except a lower respiratory rate of 10 bpm [6]. Extrapolating this rate to 18 bpm as in the present study yields 26.5 mL·h^−1^, differing by only 2 mL from the consumption in the present study. This small difference may be explained by patient uptake and leaks during endotracheal suctioning. In the same bench study, the Mirus™ system was modified by replacing its reflector by a cut out of the AnaConDa^TM^, (Sedana Medical AB, Danderyd, Sweden), another commonly used reflection system in the ICU. With this modification, desflurane consumption was less than half.

Thus, savings seem possible, and they are important because of the high greenhouse warming potential of volatile anesthetics, particularly of desflurane [30]. For the time being, we do not consider sedating ICU patients with desflurane because of economic and ecological considerations.

## 5. Conclusions

We conclude that washout and awakening times after inhaled sedation of critically ill patients with desflurane are significantly shorter than after isoflurane. Improvements in the efficiency of the anesthetic reflector could render inhaled sedation with desflurane economical and at the same time decrease its impact on climate change.

## Figures and Tables

**Table 1:** Patients characteristics before and during study sedation.

	Desflurane	Isoflurane	*p* Value
	(*n* = 10)	(*n* = 10)	
Male	8	6	0.33^1^
Age (years)	58 ± 16	66 ± 16	0.30^2^
Height (cm)	170 ± 7	174 ± 6	0.21^2^
Body mass index (kg/m^2^)	26.3 (23.5–33.4)	27.4 (25.1–30)	0.58^3^
Reason for admission:			0.62^1^
Abdominal surgery	4	5	
Trauma	1	2	
Submandibular abscess	1	0	
Necrotizing fasciitis	1	1	
Erysipelas	1	1	
Pneumonia	2	0	
Pancreatitis	0	1	
SAPS II Score ^4^ on admission	42.5 (35.0–45.0)	48 (37.0–56.0)	0.49^3^
Patients with sepsis	5	7	0.36^1^
Reason for invasive ventilation			
Airway	2	0	
Pulmonary	8	10	0.14^1^
Oxygenation index (mmHg)	197 ± 86	184 ± 46	0.69^2^
(at time of intubation)			
SOFA Score ^5^	8.5 (6.5–9.0)	8.0 (8.0–10.0)	0.21^3^
(at time of intubation)			
Duration of application of anesthetics (h)	20 ± 1	21 ± 2	0.26^2^
MAC fraction ^6^	0.45 ± 0.14	0.37 ± 0.17	0.23^2^
RASS Scores ^7^	−4 (−5; −4)	−4 (−4.9; −4)	0.97^3^
Remifentanil dose (µg/kg/min)	0.087 ± 0.072	0.082 ± 0.032	0.85^2^
(before study sedation)			
Remifentanil dose (µg/kg/min)	0.056 ± 0.033	0.037 ± 0.013	0.10^2^
(during study sedation)			
Patients breathing spontaneously	6	4	0.37^1^
(at start of study)			
Time to start breathing spontaneously	0.8 ± 0.9	0.9 ± 0.5	0.92^2^
in remaining patients (h)			
Tidal volume (mL)	580 ± 90	610 ± 130	0.58^2^
Respiratory rate (min^−1^)	18 ± 6	19 ± 6	0.83^2^

Data expressed as mean ± standard deviation or median (1st; 3rd quartile). Statistical comparison by: ^1^ chi-square test, ^2^ unpaired t-test, ^3^ Mann–Whitney-U test; ^4^ Simplified Acute Physiology Score II, ^5^ Sequential Organ Failure Assessment Score, ^6^ fraction of the age adjusted minimal alveolar concentration, ^7^ Richmond Agitation and Sedation Scale.

**Table 2:** Decrement times.

	Desflurane	Isoflurane	Number of Patients (Desflurane:Isoflurane)	*p* Value
T30%	0.1 (0.1–0.2)	0.2 (0.2–0.5)	10:10	0.034
T40%	0.2 (0.2–0.2)	0.5 (0.4–1.1)	10:10	<0.001
T50%	0.3 (0.3–0.4)	1.3 (0.4–2.3)	10:10	0.002
T60%	0.7 (0.4–0.9)	1.5 (0.8–2.9)	10:8	0.006
T70%	1.1 (0.6–1.9)	4.3 (2.0–8.2)	9:8	0.005
T80%	2.5 (2.0–5.9)	12.1 (5.1–20.2)	7:6	0.022

All times are given in minutes, median (1st; 3rd quartile). Statistical comparison using log-rank test. The 50% decrement time (T50%) was the main outcome measure of this study.

## Data Availability

The data presented in this study are available on request from the corresponding author P.D. or A.M. The data are not publicly available due to privacy restrictions.
